# Altered expression of AXL receptor tyrosine kinase in gastrointestinal cancers: a promising therapeutic target

**DOI:** 10.3389/fonc.2023.1079041

**Published:** 2023-07-04

**Authors:** Nataliya Pidkovka, Abbes Belkhiri

**Affiliations:** ^1^ Department of Health Science, South College, Nashville, TN, United States; ^2^ Department of Surgery, Vanderbilt University Medical Center, Nashville, TN, United States; ^3^ Vanderbilt-Ingram Cancer Center, Vanderbilt University Medical Center, Nashville, TN, United States

**Keywords:** GI cancers, targeted therapy, small molecule inhibitors, anti-AXL antibodies, Gas6

## Abstract

Gastrointestinal (GI) cancers that include all cancers of the digestive tract organs are generally associated with obesity, lack of exercising, smoking, poor diet, and heavy alcohol consumption. Treatment of GI cancers typically involves surgery followed by chemotherapy and/or radiation. Unfortunately, intrinsic or acquired resistance to these therapies underscore the need for more effective targeted therapies that have been proven in other malignancies. The aggressive features of GI cancers share distinct signaling pathways that are connected to each other by the overexpression and activation of AXL receptor tyrosine kinase. Several preclinical and clinical studies involving anti-AXL antibodies and small molecule AXL kinase inhibitors to test their efficacy in solid tumors, including GI cancers, have been recently carried out. Therefore, AXL may be a promising therapeutic target for overcoming the shortcomings of standard therapies in GI cancers.

## Introduction

Increasing cancer risk factors linked to emerging economy and globalization have aggravated the global cancer burden with an expected 47% increase of incidence in 2040 relative to 2020 (1). The rising disease burden caused by the malignancies of the digestive system has become one of the major public health challenges. Particularly, colorectal (10%) stomach (5.6%), esophageal (3.1%), liver (8.3%), and pancreatic (4.7%) cancers are among the most diagnosed malignancies after female breast and lung cancers (11.7% and 11.4% accordingly) (1, 2). Therefore, there is a critical need for identifying reliable molecular markers and targets for gastrointestinal (GI) oncotherapies. For the last decade, AXL receptor tyrosine kinase, also known as UFO, attracted a substantial interest in cancer biology because of the progressively accumulated data demonstrating the ability of this protein to regulate cell survival, proliferation, and motility in normal and cancer tissues (3–8). The selective overexpression of AXL in GI malignancies is associated with a poor clinical prognosis (9–11), proliferation (10, 12, 13), metastasis (14), immunosuppressive tumor microenvironment (3, 15, 16), and drug resistance (17, 18). This review provides a comprehensive update on the current research initiatives highlighting AXL as a promising therapeutic target and a novel diagnostic and prognostic marker of GI cancers. The research findings from preclinical and clinical studies on the evaluation of drugs in targeting the AXL-mediated signaling pathways in GI cancers are reviewed.

### AXL function and signaling

AXL protein (100 - 140 kDa) belongs to the receptor tyrosine kinase (RTK) subfamily of transmembrane receptors TAM, which comprises TYRO3 (19), AXL (20), and MER (21–23). Initially, AXL was identified as a transforming gene in patients with chronic myelogenous leukemia (24). Later, the names AXL (from the Greek “anexelekto”, meaning “uncontrolled”) and UFO were given concurrently to the same cDNA encoding an RTK overexpressed in human myeloid leukemia cells (25, 26) and NIH3T3 mouse fibroblasts transfected with DNA from a patient with a chronic myeloproliferative disorder (20, 27). TAM family of RTKs is characterized by a combination of two immunoglobin-like domains and fibronectin type III domains in the extracellular (N-terminal) region. AXL also has an intracellular (C-terminal) tyrosine kinase domain, which plays an essential role in signal transduction (28). The vitamin k-dependent growth arrest-specific protein 6 (Gas6) (29) serves as a high affinity ligand for AXL (21, 30, 31). Gas6 binding to AXL primes the homodimerization of receptor with another Gas6/AXL ligand-receptor complex and autophosphorylation of three tyrosine residues (32). This set of reactions initiates the recruitment of p85 subunit of phosphoinositide-3 kinase (PI3K), phospholipase C-γ (PLCγ), or growth factor receptor–bound protein 2 (Grb2) and activate the relevant downstream signaling pathways involved in survival, proliferation, or migration (33, 34). Notably, the activation of AXL is negatively regulated by the binding of its soluble form sAXL to Gas6 (34, 35). Important physiological functions of the Gas6/AXL pathway include cell migration and survival (36), adhesion (37), and suppression of apoptosis (38) in inflammatory, endothelial, and smooth muscle cells. Additionally, the Gas6/AXL signaling plays an important role in the activation of macrophages and phagocytosis (39).

### AXL expression in GI cancers (esophagus, stomach, pancreas, liver and colon)

Since genetic modifications of *AXL* gene, such as rearrangement, amplification, or mutations, are relatively rare (40, 41), the AXL functions in GI cancers are likely determined by the level of its expression. High expression of AXL has been reported in a variety of primary GI tumors and metastases and linked to poor clinical prognosis (**Table 1**) (11, 42–45). Invasive esophageal adenocarcinoma (EAC) frequently progresses from a premalignant condition, gastroesophageal reflux disease-associated Barrett’s esophagus (BE). AXL expression is linked to adverse prognosis in EAC (11) as well as poor prognosis and distant metastases in esophageal squamous cell carcinoma (43). Particularly, serial analysis of gene expression (SAGE) indicated a significant upregulation of AXL “tags” in metachronous mucosal biopsy samples obtained from a patient progressed from BE to EAC (11). Moreover, both univariate and multivariate analyses of 92 surgically resected sections of EAC demonstrated a positive correlation of AXL overexpression with decreased median survival of the patients (11). Elevated expression of AXL and p-AXL (Y779) proteins was detected by immunoblot analysis in human EAC cell lines SK-GT-4, FLO-1, and JH-EsoAd1 as compared to normal esophageal squamous epithelial cell lines (18). Immunohistochemical (IHC) staining with anti-AXL specific antibody of tissue microarrays indicated AXL overexpression in 51.8% of EAC tumors relative to normal esophageal squamous tissue specimens (18). The results of a further IHC analysis performed on tissue microarrays including 53 human EAC and 11 normal esophageal tissues revealed that AXL as well as another potentially prooncogenic molecule non-receptor tyrosine kinase c-ABL were overexpressed in 55% and 66% of EAC samples, respectively, as compared to normal tissue specimens (46). Moreover, co-overexpression of AXL and c-ABL was detected in 49% of EAC samples (46).

**Table 1:** Overview of AXL overexpression in GI neoplasms.

GI organ/type of cancer	AXL overexpression or gene amplification	References
Barrett’s esophagus/low grade dysplasia/high grade dysplasia/esophageal adenocarcinoma (EAC)	Protein (IHC), DNA (SAGE)	(11)
EAC	Protein (IHC)	(46, 47)
Esophageal squamous cell carcinoma (ESCC)	Protein (IHC)	(43)
Hepatocellular carcinoma (HCC)	Protein (IHC) Protein (WB and IHC) sAXL protein in plasma (ELISA) sAXL protein in plasma (ELISA)	(48) (44) (49) (48)
Pancreatic ductal adenocarcinoma (PDA)	Protein (IHC) Protein (IHC)	(13) (50)
Gastric cancer	mRNA (qRT-PCR), protein (IHC)	(45)
Colorectal carcinoma (CRC)	mRNA (Array), protein (IHC) mRNA (NGS), protein (IHC) DNA (FISH), protein (IHC)	(9) (42) (51)

IHC, immunohistochemistry; WB, western blotting; ELISA, enzyme-linked immunosorbent assay; qRT-PCR, quantitative real-time PCR; NGS, next generation sequencing; FISH, fluorescence in situ hybridization; sAXL, soluble AXL protein; SAGE, serial analysis of gene expression.

High mRNA and protein expression of Gas6 and AXL has been reported in human gastric cancer cell lines and tissues (45). Notably, Gas6 expression was significantly correlated with lymph node metastases (45).

The immunohistochemical evaluation of expression of AXL protein in a panel of 99 archival pancreatic cancers revealed AXL expression in 54 out of 99 specimens (55%); and positive AXL expression in pancreatic cancer was significantly associated with lymph node metastases and a shorter median survival (12 as opposed to 18 months) as compared to AXL-negative tumor samples (50). Frequent overexpression of both molecules, Gas6 and AXL, has been detected in Pancreatic Ductal Adenocarcinoma (PDA) cells and was linked to a poor prognosis in patients with stage II PDA (13).

Additionally, high AXL mRNA and protein expression levels were associated with poor overall survival in early-stage colorectal cancer (CRC) tissues (42). Particularly, the statistical analysis of CRC microarray dataset, available through the Gene Expression Omnibus (GEO) (52), showed a significant association between high AXL mRNA expression and decreased disease-specific survival in a cohort of 177 patients diagnosed with an early-stage (stage II/III) CRC (42). Furthermore, AXL overexpression in colorectal adenocarcinoma as compared to normal colon tissues was demonstrated by IHC in tissue microarray resection specimens of primary tumors collected from 509 patients with colorectal adenocarcinoma (stage I-IV) at the National University Hospital of Singapore between 1990 and 1999 (42). Likewise, the overexpression of AXL and GAS6 was shown by IHC in 76,7% and 73.5%, respectively, in 223 human CRC specimens, while the amplification of *AXL* gene was detected by fluorescence *in situ* hybridization (FISH) in 8 out of 146 cases (5,4%) of CRC samples (51). The increased expression of AXL and GAS6 proteins was correlated with less differentiated histological grading, tumor stage and lymph nodes involvement (51). Given that majority of patients with high-risk stage II/III CRC tend to relapse (53) and progress to the advanced stages, AXL could be used as a prognostic biomarker for the distal part of GI tract.

While all known methods to evaluate AXL expression include tissue extraction, in some forms of hepatic neoplasm, it is possible to assess clinical outcome by evaluating plasma levels of soluble AXL (sAXL). It is an 85 kDa N-terminal product of extracellular ADAM metalloproteases-dependent proteolytic cleavage of AXL, which has GAS6 ligand-binding abilities and serving as a decoy receptor (26, 54, 55). This circulating sAXL has a promising potential as a specific serum marker of cirrhosis and early stages of hepatocellular carcinoma (HCC) (49). It has been documented that serum concentrations of sAXL were elevated at early (82.57 ng/mL) and later stages (114.50 ng/mL) of HCC in comparison with healthy controls (40.15 ng/mL) (49). Notably, sAXL levels were not altered in patients with chronic liver disease, liver adenomas and cholangiocarcinomas (49). These data suggest that elevated concentration of sAXL is a valuable biomarker of liver neoplastic transformation that could be noninvasively detected in plasma. In another study analyzing the diagnostic potential of this molecule, sAXL levels were evaluated in 311 HCC and 237 control serum samples collected from clinical centers in Europe and China (56). Average concentrations of sAXL were significantly higher in the serum of HCC patients (18.575 ng/mL) as compared to healthy (13.388 ng/mL) or cirrhotic (12.169 ng/mL) controls (56). Levels of sAxl remained unchanged in the serum of individuals diagnosed with primary ovarian, colorectal and breast carcinomas, or secondary colon-derived hepatic malignancies. Consequently, the soluble form of AXL was suggested as highly specific and accurate diagnostic marker for alpha-fetoprotein-negative HCC patients (56). Additionally, sAXL was proposed as a biomarker for early diagnosis of PADC based on the studies Martinez-Bosch, N. et al, 2022, which demonstrated increased sAXL levels in plasma of PDAC group as compared to healthy controls or chronic pancreatitis (CP) patients. Immunohistochemical analysis revealed higher protein expression in tissues samples obtained from PDAC and precancerous lesions as compared to CP or healthy control specimens. The immunohistochemistry data was confirmed by RNA expression analysis from TCGA database. It was noted that patients with high levels of AXL have a lower overall survival. Importantly, ROC statistical analysis of the plasma levels of sAXL, GAS6, or CA19-9 (a marker of pancreatic cancer) in two studied cohorts revealed that sAXL outperformed CA19-9 for discriminating between CP and PDAC (57). The data showing increased AXL expression in GI cancer tissues are summarized in **Table 1**.

The mechanisms leading to AXL overexpression are tissue-specific and may vary depending on local tissue microenvironment. Irrespective of AXL localization, the alteration of patterns of this molecule expression could be considered a hallmark of GI carcinogenesis. The specific roles of AXL in the alteration of basic cell functions in GI cancers are discussed below.

### Proliferation and survival

Initial steps in carcinogenesis are associated with uncontrolled proliferation and survival of transformed or cancer stem cells (58). Gas6-AXL signaling pathway has been shown to enhance cell survival and suppress apoptosis in gastric cancer cells through activation of the AKT pathway (45). In another study, YAP-dependent cell survival and proliferation required AXL expression and activation of ERK1/2 signaling cascade in human HCC (59). Additionally, the proliferation of a metastatic HCC *in vitro* and *in vivo* was markedly suppressed by tunicamycin-induced de-glycosylation and downregulation of AXL (60).

In pancreatic ductal adenocarcinoma, the upregulation of AXL has been associated with a poor clinical prognosis and increased cell proliferation (12, 13), while stable knockdown of AXL resulted in a significant reduction in cell viability and anchorage-independent growth in pancreatic cancer cells (50). In a preclinical study, treatment with S49076, an ATP-competitive tyrosine kinase inhibitor of MET, AXL, and FGFR1, significantly inhibited colony formation in soft agar by HCC cells overexpressing AXL and FGFR2 (61). The lack of sensitivity to S49076 in the same cell lines cultured in monolayer (61) suggests a key role of AXL in extracellular matrix anchorage-independent growth and survival. Indeed, the results of currently available preclinical and clinical studies suggest that the primary roles of AXL and other TAM RTKs may be mostly related to the mechanisms of survival, motility, and drug resistance rather than functioning as oncogenic drivers (62, 63).

### Epithelial–mesenchymal transition and metastasis

The epithelial–mesenchymal transition (EMT) is a process by which epithelial cells undergo morphological and functional changes towards a mesenchymal phenotype (64). During this process of trans-differentiation, epithelial cells lose their polarity as well as cell-cell adhesion properties and acquire characteristics of mesenchymal stem cells (65). Cancer cells detach from the primary tumor location, migrate through the extracellular matrix, and intravasate into blood vessels, promoting metastases (66, 67). Importantly, residual metastatic disease from the primary tumor remains the major reason of recurrence and greater than 90% of cancer-related death (68). In cancers of the digestive system, AXL overexpression in tumors and metastases indicates adverse clinical prognosis in patients (9, 10, 42–44, 51, 69).

EMT associated with intrahepatic metastasis is a typical feature of HCC (48). Several studies in HCC have demonstrated elevated levels of AXL transcript and protein in association with EMT (48, 56, 59). For example, AXL mRNA overexpression and correlation with EMT has been documented in 28 HCC cell lines and 373 RNA-seq tissue datasets in comparison with cirrhotic and normal liver samples (70). A crucial role of AXL in transforming growth factor beta (TGF-β)-dependent HCC progression was proposed based on the studies revealing upregulation and activation of AXL in EMT-altered hepatoma cells (48). At the same time, AXL activation by Gas-6 increased TGF-β1 mRNA, while AXL knockdown dramatically reduced resistance to TGF-β-dependent growth inhibition by abrogating invasion and trans-endothelial migration of mesenchymal HCC cells (48). Notably, AXL overexpression triggered metastatic colonization of epithelial hepatoma cells *in vivo.* Immunohistochemical analysis of AXL expression in tumor tissues collected from 133 HCC patients demonstrated a correlation of increased AXL expression with advanced tumor stages, augmented vessel invasion of HCC cells, elevated risk of cancer relapse after liver transplantation, and a poor clinical prognosis (48). One of the most severe metastatic complications in HCC is portal vein tumor thrombus (PVTT). It has been shown that co-implantation of human umbilical vein endothelial cells (HUVECs) overexpressing AXL with HCC cells in xenograft nude mice and patient-derived xenograft (PDX) nude mice substantially enhanced tumor growth, hepatic metastasis, and vessel metastasis of HCC (71). These effects were suppressed by an AXL inhibitor R428, also known as BGB324 or bemcentinib (71).

Several studies suggested AXL as a potential therapeutic target in pancreatic cancer (13, 50, 72, 73). Thus, the immunohistochemical assessment of 99 pancreatic cancer specimens revealed a higher number of lymph node metastases and a shorter median survival of patients with AXL-positive tumors (12 versus 18 months) in contrast to the AXL-negative group (50). Stable knockdown of endogenous AXL in pancreatic cancer cells resulted in a significant decrease of mRNA levels of matrix metalloproteinase (MMP)-9 and EMT-associated transcription factors twist, snail, and slug (50). Moreover, AXL knockdown cells exhibited reduction in cell viability, migration, and invasion (50). The role of AXL signaling in progression and metastasis of pancreatic cancer was confirmed in a study using low-dose warfarin, a vitamin K “antagonist” to inhibit Gas6-dependent AXL activation (72). Treatment with low-dose warfarin reduced AXL-mediated human pancreatic cancer cells migration, invasiveness, and proliferation, while increasing apoptosis and sensitivity to chemotherapy. Additionally, warfarin decreased primary tumor growth and suppressed metastases in a murine model of pancreatic ductal adenocarcinoma (PDAC) (72). On a molecular level, low-dose warfarin treatment blocked TGFβ-induced expression of AXL, and markedly reduced expression levels of mesenchymal markers, Zeb1 and nuclear β-catenin in Panc-1 pancreatic epithelioid carcinoma cell line. Consistently, warfarin inhibited expression of vimentin and increased levels of E-cadherin in AXL-positive Panc-1 xenografts (72).

AXL signaling axis is also implicated in EMT of GI cancers. For instance, high levels of Gas6 and AXL mRNA and proteins were revealed in human gastric cancer cell lines and tissue samples, and Gas6 expression was significantly correlated with metastases to lymph nodes (45). The *in vitro* experiments using recombinant Gas6 and a decoy-receptor of AXL showed that activation of Gas6-AXL signaling axis leads to the inhibition of apoptosis and exacerbation of AKT-dependent survival and invasion of gastric cancer cells (45). In another study, inhibition of AXL-NF-κB signaling pathway by ursolic acid markedly inhibited cell migration and reduced the expression of mesenchymal markers and EMT-related transcription factors in gastric cancer cells and xenografts (74). In EAC cells, genetic silencing of AXL attenuated invasion, migration, and *in vivo* engraftment. Furthermore, pharmacological inhibition of AXL with small molecule agent R428 has shown similar functional effects in EAC cells (11). Our studies in EAC cell lines demonstrated that increased expression of AXL facilitates peripheral distribution of lysosomes leading to activation of cell invasion signaling cascade through the regulation of cathepsin B secretion (75). Besides, we found that these processes were caused by extracellular acidification because of AXL-induced secretion of lactate through AKT-NF-κB–dependent synthesis of lactate transporter MCT-1 (75).

Recently, dual inhibition of TGFβ and AXL signaling pathways was proposed as a novel therapy for human colorectal adenocarcinoma with mesenchymal phenotype (CMS4), a very aggressive CRC characterized by resistance to standard chemotherapies, low survival rate and high risk of recurrence (76, 77). In fact, overexpression of AXL and TGFβ receptors in CMS4 tumors correlated with higher risk of post-surgical relapse in stage II/III CRC and decreased survival (76). In CRC cell lines, treatment with the TGFβ inhibitor, galunisertib, and the AXL inhibitor, R428, markedly reduced colony formation and migration of cancer cells, and demonstrated potent anti-tumor activity in 3D spheroid cultures obtained from individuals with advanced CRC (76). Additionally, multitarget tyrosine kinase inhibitor (TKI) cabozantinib and AXL/c-MET selective inhibitor R428 both decreased AXL phosphorylation and TGFβ-induced E-cadherin expression (marker of EMT), cell viability, migration, and tumor growth in esophageal squamous cell carcinoma (ESCC) cells and xenograft models (78).

Interestingly, AXL expression was upregulated by Long non-coding RNA (lncRNA) CALIC in complex with RNA-binding protein hnRNP-L in colon cancer cells, while knockdown of either CALIC or AXL inhibited metastases *in vivo* (14). Application of AXL expression as a marker of poor prognosis and a crucial mediator of cell invasion was proposed for early-stage CRC, specifically in the adjuvant disease in the cases of unsuccessful EGFR/VEGF–targeted therapies (42).

### AXL in angiogenesis

Angiogenesis is the formation of new blood vessels that often promote tumor growth and progression. AXL regulates many angiogenic activities such as proliferation and migration of vascular smooth muscle cells (VSMCs) and endothelial cells (ECs) (36), tube formation *in vitro* and angiogenesis *in vivo* (3). Vascular endothelial growth factor (VEGF) is secreted in high levels by most types of cancer cells (79). Proliferation and migration of VSMC are necessary for tumor angiogenesis (80). In fact, VSMCs express Gas6, and exogenous Gas6 promotes proliferation and migration of VSMCs (36). AXL also is expressed by tumor stromal cells, including ECs (7, 81). Knockdown of AXL or Gas6 expression markedly inhibited migration of HUVECs, while AXL overexpression enhanced cell growth and tubes formation (3). Notably, overexpression of AXL expression was observed in HCC-tumor-derived endothelial cells (TECs), although not in the tumor cells of HCC patients with portal vein tumor thrombus (PVTT) type of metastases. These data were associated with poor overall survival and disease-free survival of HCC patients with PVTT (71). Moreover, elevated expression of AXL was associated with the expression of a marker of endothelial cells CD 31 *in vitro* and *in vivo* (71).

Interestingly, Axl*-*null mice exhibited an impaired angiogenesis and vascular permeability in response to VEGF-A treatment (82). Therefore, it has been proposed that AXL could be one of the essential mediators of VEGF-A-dependent activation of pro-angiogenic PI3K/AKT signaling pathway (82). Accordingly, using AXL inhibitors in addition to anti-VEGF therapeutics could be an effective strategy targeting neovascularization in GI cancers.

### AXL in the immune response to tumors

Inflammation is a one of the hallmarks of carcinogenesis, and it has been proven that chronic inflammation caused by autoimmune gastritis and *Helicobacter pylori* infection increases a risk of developing gastric cancer (83). In fact, more than 90% of gastric adenocarcinomas originate from epithelial cells of gastric mucosa because of chronic inflammation (83). Tumor intrinsic and immunosuppressive mechanisms contribute to conventional chemotherapy resistance (84). TAM family of RTKs, including AXL, are key regulators of immune response (85). Following their activation by Gas6 ligand – activator of all TAM family members, and Protein S ligand -activator of both MER and TYRO3, these receptors promote the resolution of inflammation by suppressing activation of cells of the innate immune system (86). Remarkably, studies on *Tyro3* ^−/−^ *Axl* ^−/−^ *Mer* ^−/−^ triple mutant mice (*TAM* TKOs) have demonstrated that loss of function of the three receptors, *T*yro3, *A*xl, and *M*er, dysregulates the immune system, presented by a severe lymphoproliferative disorder accompanied by a broad-spectrum autoimmune disease (39, 87). In a cancer setting, TAM receptors regulate the initiation and progression of tumorigenesis and, simultaneously, the anti-tumor functions of immune cells (85). Tumor progression is considerably affected by the tumor microenvironment (TME), comprised of all host cells and tissue components surrounding the cancer cells (88). On the other hand, programmed cell death through apoptosis maintains tissue homeostasis and prevents oncogenic transformation. Clearance of cell debris is the last stage of apoptosis. Uncleared products of this process might induce necrosis, thereby promoting inflammation and autoimmunity (89). Externalized phosphatidylserine (PS) acts as “eat-me” signal on apoptotic cells, stressed cells, exosomes, and liposomes. Importantly, endogenous ligands Gas6 and Protein S link externalized PS molecules with TAMs, activating those RTKs and promoting clearance of apoptotic cells (90, 91).

Studies on animal models have shown that TAM family receptors are involved in the clearance of apoptotic cells by macrophages and dendritic cells (DC) (92, 93). In fact, treatment with dextran sulfate sodium (DSS) salt blocked clearance of apoptotic neutrophils in the lamina propria of large intestine and promoted colitis in Axl^−/−^Mer^−/−^ double mutant mice (94). The Authors demonstrated that the observed inflammatory phenotype is associated with the knockout of *Axl* and *Mer* genes in radioresistant population of macrophages residing specifically in the intestinal tissues, while loss of Axl and Mer in the radiosensitive bone marrow–derived hematopoietic cells was not linked to exacerbated colitis (94). As such, AXL and MERTK inhibitors might induce adverse effects at the systemic level, and physiological effects of the alteration of AXL and MERTK signaling could be highly tissue-specific and depend on tumor microenvironment.

Tumor-associated macrophages are abundant in the TME and contribute to immunosuppression and tumor progression (92). In human and murine macrophage cultures, AXL activation has been shown to mediate Interferon α induction of Twist, a transcriptional repressor of inflammatory cytokine tumor necrosis factor α (TNFα) (95). Altering AXL expression and downstream activation of Twist highlights a promising approach to control inflammation, which is the hallmark of oncogenesis. In several studies, activation of TAM receptors not only decreased severe inflammatory responses (96), but also induced efferocytosis and macrophage polarization towards a pro-tumor M2-like phenotype, accompanied by the increased production of immunosuppressive cytokines (92, 97–99). AXL induced TANK binding kinase 1 (TBK1)-NF-κB signaling pathway and innate immune suppression in the TME in pancreatic cancer (73), while inhibition of AXL with small molecule R428 enhanced immune stimulatory microenvironment (73). Immune checkpoint blockade (PD-1) is a novel popular approach in cancer immunotherapies. Unfortunately, some of the tumors are resistant to PD-1 inhibitors and considered to be immunologically “cold,” because of the lack of tumor antigen-specific primed cytotoxic T cells (99). It has been shown that Sitravatinib, a broad-spectrum tyrosine kinase inhibitor (TKI) targeting MET, TAM, and members of VEGFR, platelet-derived growth factor receptor (PDGFR), and Eph families is highly effective in various cancer models, including CT1B-A5, an isogenic pancreatic cancer cell line, that could be partially attributed to altering the TME and restoring the efficacy of immune checkpoint blockade (PD-1) (99). Therefore, AXL is one of the major drivers of immune suppression in the TME. Although AXL-mediated pathway is an attractive candidate for inhibition in GI cancers to reverse the immunosuppressive TME, further investigations are needed as this therapeutic approach may cause adverse systemic effects like inflammation and autoimmunity.

### AXL in resistance to anti-cancer therapies

One of the major problems of anti-cancer therapies is that many cancers are initially responsive to treatment, but ultimately develop drug resistance that may lead to an unfavorable clinical outcome (100). AXL overexpression in GI cancers has been associated with resistance to both targeted and non-targeted anti-cancer therapies. Particularly, EAC is characterized by resistance to chemotherapy and poor prognosis (18). Notably, AXL overexpression has been shown to mediate resistance to epirubicin by upregulation of c-MYC transcription *via* AKT-β-catenin signaling pathway in EAC cells (17). Additionally, AXL has been proposed as a promising therapeutic target to sensitize GI cancers to DNA-damaging chemotherapy drugs. In fact, genetic silencing of endogenous AXL abrogates cisplatin resistance through inhibition of the pro-apoptotic c-ABL/p73β signaling pathway in human EAC cells (18). AXL expression also promotes resistance to TNF-related apoptosis-inducing ligand (TRAIL) mediated by death receptor 5 (DR5) activity in EAC cells (47). Specifically, AXL and DR5 protein interaction blocks the recruitment of caspase-8 to the death-inducing signaling complex (DISC), resulting in enhanced cell survival, and decreased apoptosis. Sensitivity to TRAIL was restored in EAC cells after genetic silencing of endogenous AXL (47).

Resistance to the chemotherapy drug gemcitabine in PDAC was attributed to the function of YWHAZ/14-3-3 zeta/delta (14-3-3ζ) protein, which was isolated from monocyte-derived macrophage cultures (101). In mice bearing orthotopic PDAC xenografts, the antitumor activity of gemcitabine was significantly enhanced by pharmacological inhibition of AXL, which is a binding protein partner of 14-3-3ζ (48, 101). Therefore, it was suggested that apoptosis induced by chemotherapy might in turn activate a survival pathway through 14-3-3ζ/AXL and AKT phosphorylation cascade (101). Treatment of PDAC cells with BGB324, a selective small molecule inhibitor of AXL, promotes epithelial differentiation, stimulatory immune microenvironment, and high expression of nucleoside transporters, enhancing the response to gemcitabine (73). Of note, BGB324 treatment also improved survival and gemcitabine sensitivity in mice with advanced PDAC (73).

Based on a large body of evidence (9, 102, 103), cancer progression is frequently associated with acquired resistance to the inhibitors of EGF receptor (EGFR) mediated by the enhanced AXL expression as a bypass mechanism. For instance, increased AXL mRNA levels were found in 5 out of 7 CRC patients following anti-EGFR therapy (9). Moreover, resistance to anti-EGFR drugs accompanied by high AXL expression was demonstrated in three-dimensional CRC cell cultures derived from an AXL-positive, RAS wild-type patient after anti-EGFR treatment (9). Furthermore, AXL overexpression in CRC cell lines led to the resistance to EGFR inhibition. The role of AXL in EGFR inhibition resistance was established by analysis of AXL expression in tumor xenograft mice and in CRC patients after anti-EGFR treatment (9). Overexpression and activation of AXL upregulates PI3K/mammalian target of rapamycin (mTOR) and MAPK signaling pathways, enhancing cell survival, cell growth, invasion, and migration (104). Particularly, AXL mediates resistance to PI3Kα inhibition through activation of EGFR/PKC/mTOR cascade in head and neck (H&N) carcinomas as well in ESCC (105). The study suggested simultaneous EGFR and PI3Kα inhibition as a prospective therapeutic approach to overcome AXL-dependent resistance to PI3Kα inhibitors in patients with esophageal and H&N squamous cell carcinomas (105).

AXL plays a major role in promoting resistance to several common chemotherapeutics and targeted anti-cancer therapies. For example, treatment with AXL inhibitor S49076 markedly decreased tumor resistance to bevacizumab, a VEGF/VEGFR blocker, in a colon carcinoma xenograft model and attenuated colony formation of FGFR1/2- and AXL-positive hepatocarcinoma cells (61). In addition, cabozantinib, a dual inhibitor of MET and AXL, decreased cell growth in both *in vitro* and *in vivo* models of HER2-amplified gastric cancer with acquired resistance to afatinib, a pan-HER inhibitor (106). Studies in ESCC cell model have demonstrated a synergistic effect of combinatory treatment with HER2 inhibitor lapatinib and AXL inhibitor foretinib (43). Notably, in esophageal tissue of patients diagnosed with operable primary ESCC, the cumulative expression of AXL and HER2 was associated with unfavorable clinical outcome (43). Therefore, drug resistance to lapatinib could be potentially overcome by the inhibition of AXL. In HCC cell lines, AXL inhibition with RNA-interference or R428 compound improved sensitivity to sorafenib associated with increased phosphorylation level of AXL (70). Elevated level of AXL expression and its activation have been implicated in the resistance to imatinib in gastrointestinal stromal tumors (GIST) (107). Since GI cancer mesenchymal cells exhibit high levels of AXL expression, this RTK is potentially a promising therapeutic target for overcoming chemoresistance and improving the efficacy of current cancer therapies.

### Targeting AXL in GI cancers

With the development of personalized medicine and targeted therapy, including tyrosine kinase inhibitors, GI cancer treatment continues to progress. Preclinical studies demonstrated that small-molecule TAM inhibitors, such as R428 (73) and RXDX106 (108), display anti-cancer activity in GI organs. As AXL has been associated with various stages of carcinogenesis and the inhibition of its expression and activity demonstrated promising results, AXL-specific inhibitors are currently being evaluated in clinical studies. BGB324 (BerGenBio); also known as R428 (Rigel Pharmaceuticals), is an oral selective small molecule AXL inhibitor that is currently being investigated in phase II clinical trials of pancreatic neoplasms (**Table 2**). BGB324 enhanced the efficacy of gemcitabine in preclinical studies *in vivo* through the stimulation of immune cellular response, expression of nucleoside transporters and promotion of epithelial cells differentiation in PDAC (73). Additionally, BGB324 is currently being tested in clinical trials as a monotherapy and in combination with chemo-, targeted-, and immunotherapy in various cancers (acute myeloid leukemia (AML), NCT02488408; non-small cell lung cancer (NSCLC), NCT02424617, NCT02922777; melanoma, NCT02872259). Particularly, combinations with nab-paclitaxel, gemcitabine, or cisplatin have shown encouraging results of clinical activity in patients with metastatic pancreatic cancer (NCT03649321).

**Table 2:** Current clinical trials testing AXL-targeted agents in GI cancer patients.

Intervention	Primary target	Condition	Co-treatment/comparator	Clinical trial	Identifier
BGB324	Inhibitor of AXL kinase	Pancreatic cancer	Nab-paclitaxel Gemcitabine Cisplatin	Phase 1 Phase 2	NCT03649321
AVB-500	AXL decoy soluble receptor, binds GAS6	Phase 1 Safety and Tolerability Study	Placebo	Phase 1	NCT03401528
AVB-500	AXL decoy soluble receptor, binds GAS6	Pancreatic Adenocarcinoma	Nab paclitaxel Gemcitabine	Phase 1 Phase 2	NCT04983407
BA3011	Conditionally active biologic anti-AXL antibody drug conjugate	Advanced Solid Tumor (Phase 1) Solid Tumor	PD-1 inhibitor	Phase 1 Phase 2	NCT03425279
Cabozantinib	Small molecule inhibitor of multiple receptor tyrosine kinases including MET, VEGFR 1, 2 and 3, AXL, and RET	Gastric Cancer Esophageal Adenocarcinoma Hepatocellular Carcinoma Colorectal Cancer	Durvalumab anti-(Programmed cell death protein 1 (PD-L1) inhibitor Tremelimumab	Phase 1 Phase 2	NCT03539822
Cabozantinib		Hepatocellular Carcinoma	Placebo	Phase 3	NCT01908426
Cabozantinib		Hepatocellular Carcinoma		Phase 2	NCT04316182
Cabozantinib		Hepatocellular Carcinoma Recurrent Cancer Liver Transplant		Phase 2	NCT04204850
Cabozantinib		Hepatocellular Carcinoma	Sorafenib Atezolizumab	Phase 3	NCT03755791
Cabozantinib		Malignant Solid Tumor		Phase 2	NCT04116541
SLC-391	Inhibitor of AXL	Solid Tumor		Phase 1	NCT03990454
TP-0903	Inhibitor of AXL	Advanced Solid Tumors EGFR Positive Non-small Cell Lung Cancer Colorectal Carcinoma Recurrent Ovarian Carcinoma BRAF-Mutated Melanoma		Phase 1	NCT02608268
Warfarin	Inhibits AXL activation, Vitamin K agonist	Pancreatic Cancer		Withdrawn	NCT03536208
MGCD516	c-Kit, PDGFRα/β, TAM, VEGF	Advanced Cancer		Phase 1	NCT02219711
BPI-9016M	Inhibitor of MET/AXL kinases.	Solid tumors		Phase 1	NCT02478866
Crizotinib	a small molecule directed to vascular endothelial growth factor receptors, MET and AXL	Hematologic Cancers Solid Tumors Metastatic Cancer		Phase 2	NCT02034981
INCB081776	Inhibitor of AXL and Mer that blocks TAM	Advanced Solid Tumors	INCMGA00012	Phase 1	NCT03522142

AVB-500 (AVB-S6-500, Batiraxcept; Aravive, Inc.) is a novel high affinity Fc-sAXL fusion protein, which acts as an AXL decoy receptor by binding Gas6 and blocking AXL signaling (109). Preclinical data demonstrated inhibition of Gas6-induced AXL and Src phosphorylation, tumor vessel density, tumor growth, and metastatic burden in renal cell carcinoma (6, 110, 111) and ovarian cancer (109, 112) in response to treatment with AVB-500. Compared with chemotherapy alone, AVB-500 in combination with carboplatin and/or paclitaxel attenuated ovarian cancer cell survival *in vitro* and tumor growth *in vivo* (112). AVB-500 is currently investigated in Phase I/II clinical trials for patients with platinum-resistant or recurrent ovarian, fallopian tube, or peritoneal cancers as a combination therapy (Clinical Trial Identification #s: NCT03639246, NCT04019288) (**Table 2**). Also, a Phase 1b/2 study of AVB-500 safety and efficacy as a monotherapy or in combination with cabozantinib or nivolumab in patients with advanced or metastatic clear cell renal cell carcinoma is in progress (Clinical Trial Identification # NCT04300140). Three other studies are currently active and recruiting patients for ovarian cancer, advanced urothelial carcinoma, and pancreatic adenocarcinoma to assess AVB-500 efficacy as a combination therapy either with paclitaxel, or cabozantinib, or nab-paclitaxel/gemcitabine, correspondingly (Clinical Trial Identification #s: NCT04729608, NCT04004442, NCT04983407) (113).

A promising approach using a conditionally active biologic (CAB) AXL-targeted antibody drug conjugate BA3011 CAB-AXL-ADC, BioAtla, LLC, **Table 2**, NCT 03425279) alone and in combination with a PD-1 inhibitor Nivolumab in patients with advanced solid tumors is currently getting tested in Phase I, and in adult and adolescent patients with advanced, refractory sarcoma is investigated in Phase II. BA3011 is a product of fusion of anti-AXL antibodies with anti-mitotic compound monomethyl auristatin E (MMAE). The binding of antibody part of BA3011 to AXL initiates intracellular translocation of antibody-drug conjugate (ADC) complex followed by the release of MMAE ultimately leading to cancer cell death. Patients with advanced solid tumors, including NSCLC, prostate cancer, and pancreatic cancer are currently recruited.

Cabozantinib (Cabometix, XL184, BMS-907351, Cabometyx™, BMS-907351) is an oral small molecule TKI that targets AXL, c-Met and VEGFR (**Table 2**). This inhibitor has been preclinically investigated in ESCC (78) and liver cancer (114, 115). Currently, the evaluation of cabozantinib in combination with durvalumab (anti-programmed cell death protein 1 (PD-L1) inhibitor) in patients with advanced gastroesophageal adenocarcinoma, gastric cancer, hepatocellular carcinoma, and colorectal cancer is undergoing phase I/II open label, multi-cohort trial to determine safety, tolerability, and efficacy of the treatment (Clinical Trial Identification #: NCT03539822). The investigators propose that cabozantinib in combination with checkpoint-based immunotherapeutics like durvalumab will result in synergistic effect by altering the TME. Cabozantinib has been clinically approved for patients with sorafenib-resistant HCC (116) (Clinical Trial Identification #: NCT01908426). The data from the randomized phase III CELESTIAL trial revealed a significant improvement in progression-free survival and overall survival *vs*. placebo in a cohort of patients with previously treated advanced HCC (116). The patients who were not included in CELESTIAL trial are currently enrolled in another trial evaluating the therapeutic effect of cabozantinib in the patients with HCC intolerant to sorafenib treatment or first line treatment different from sorafenib (Clinical Trial Identification #: NCT04316182, Phase II). Another trial at stage 2 is ongoing to determine the outcome of cabozantinib treatment in patients with recurrent HCC and who had received a liver transplant as a part of a previous therapy (Clinical Trial Identification #: NCT04204850). Safety and efficacy of the treatment combination of cabozantinib and atezolizumab in comparison with the standard care (treatment with sorafenib) in patients with advanced HCC, who have not received prior systemic anti-cancer treatment, is being investigated in Phase III clinical trial (NCT03755791). The clinical benefits of cabozantinib in a cohort of patients with metastatic disease or unresectable locally advanced malignancy are being assessed as a part of MegaMOST clinical study (NCT04116541).

The clinical study of SLC-391 (SignalChem Lifesciences Corporation), a novel, potent and selective small molecule inhibitor of AXL, is currently ongoing in Canada, and recruiting patients with solid tumors to determine safety and tolerability of the drug (NCT03990454). Notably, the clinical outcome of SLC-391 in combination with the anti-PD-1 therapy pembrolizumab (Keytruda^®^) will be evaluated in SKYLITE trial, a phase II study for patients with NSCLC carried by Merck (MSD) and British Columbia-based SignalChem Lifesciences. TP-0903 (Sumitomo Dainippon Pharma Oncology, Inc), a novel oral inhibitor of AXL kinase that reverses the mesenchymal cancer phenotype, is currently investigated in Phases 1a/1b clinical trial for advanced solid tumors (Clinical Trial Identification #: NCT02729298). Commonly used anticoagulant Warfarin prevents the Gas6 interaction with externalized phosphatidylserine on the surface of apoptotic cells and cell debris through inhibition of vitamin K-dependent gamma-carboxylation of the γ-carboxyglutamic acid-rich (Gla) domain of Gas6 (117). A preclinical study reported that low-dose warfarin blocks the progression and spread of pancreatic cancer (72). A Phase I study of the effect of escalating doses of Warfarin on circulating biomarkers of AXL pathways (phosphoGas6 and sAXL) in patients with pancreatic adenocarcinoma (**Table 2**, Clinical Trial Identification #: NCT03536208) was initiated in 2019, but the study was withdrawn in 2021 because of lack of accrual.

MGCD516 (Sitravatinib, Mirati Therapeutics Inc.) is a small molecule spectrum selective TKI of several closely related receptor tyrosine kinases, including TAM and members of the VEGFR, PDGFR, DDR2, TRK and Eph families (118, 119). Anti-tumorigenic and anti-angiogenic activities of MGCD516 have been demonstrated in preclinical models of soft tissue sarcoma (119) and metastatic models of anti-angiogenic therapy resistance (118). Additionally, MGCD516 treatment enhances the immune checkpoint blockade by lowering the number of tumor-associated immunosuppressive myeloid cells and expanding the populations of CD4+ T cells and proliferating CD8+ T cells in the TME (99). MGCD516 therapy is currently investigated in patients with advanced solid tumors (**Table 2**, Clinical Trial Identification #: NCT02219711).

BPI-9016M (Betta Pharmaceuticals Co., Ltd.), is a novel highly potent dual-target inhibitor of c-Met/AXL. Preclinical studies in a lung adenocarcinoma model demonstrated strong activity of BPI-9016M *in vitro* and *in vivo* against c-Met/AXL kinases and their downstream pathways, leading to reduced tumor cell growth, migration, and invasion (120). A clinical trial at Phase I (NCT02478866) is currently assessing pharmacokinetics, safety, and anti-tumor activity of the inhibitor in patients with advanced solid tumors (121). Crizotinib (XALKORI^®^, PF-02341066, Pfizer Inc.) is a multitargeted, ATP-competitive, small molecule and orally available tyrosine kinase inhibitor that inhibits c-Met, AXL, ALK, and Ron (113). Preclinically, this compound reduced cell growth and induced apoptosis in human gastric carcinoma cells (122). Additionally, crizotinib in combination with mitomycin C increased apoptosis in CRC (123). A Phase II clinical trial is ongoing for patients with hematologic cancers, solid tumors, and metastatic cancer to determine the efficacy and the safety of crizotinib in 23 cohorts of patients with identified activating molecular alterations in the crizotinib target genes (**Table 2**, Clinical Trial Identification #: NCT02034981). Overall, there are 168 clinical studies associated with crizotinib in the ClinicalTrials.gov database. INCB081776 as a monotherapy or in combination with INCMGA00012 is undergoing Phase I trial for the safety and tolerability, pharmacokinetics, pharmacodynamics, and early clinical activity in patients with advanced solid tumors (Clinical Trial Identification #: NCT03522142). AXL-targeted therapies either as particular agents or in combination with conventional chemotherapy or other small molecule inhibitors have a promising opportunity to increase the survival rate of cancer patients. Nonetheless, more studies of AXL signaling pathways and physiological effects of their alteration are essential to identify the specific cohorts of patients who would be more responsive to the treatments with fewer adverse effects.

Chimeric antigen receptor (CAR)-T cell therapy targeting the B-cell antigen CD19 has proven clinically very successful in hematologic cancers [Clinical Trial Identification #: NCT02435849, NCT02445248, and NCT02348216 (124, 125)]. However, the development of CAR-T cell therapies for solid tumors has been slow because of the unique challenges associated with tumor microenvironment (126, 127). Preclinical studies indicated that CAR-T cell therapy targeting AXL induced *in vitro* cytotoxicity in triple negative breast cancer cells (TNBC) and reduced tumor growth in a TNBC xenograft mouse model (128). Preclinical and clinical studies are needed to investigate AXL-CAR-T cell therapy approach in GI cancers with high AXL expression.

## Conclusions and future perspectives

**Figure 1:**
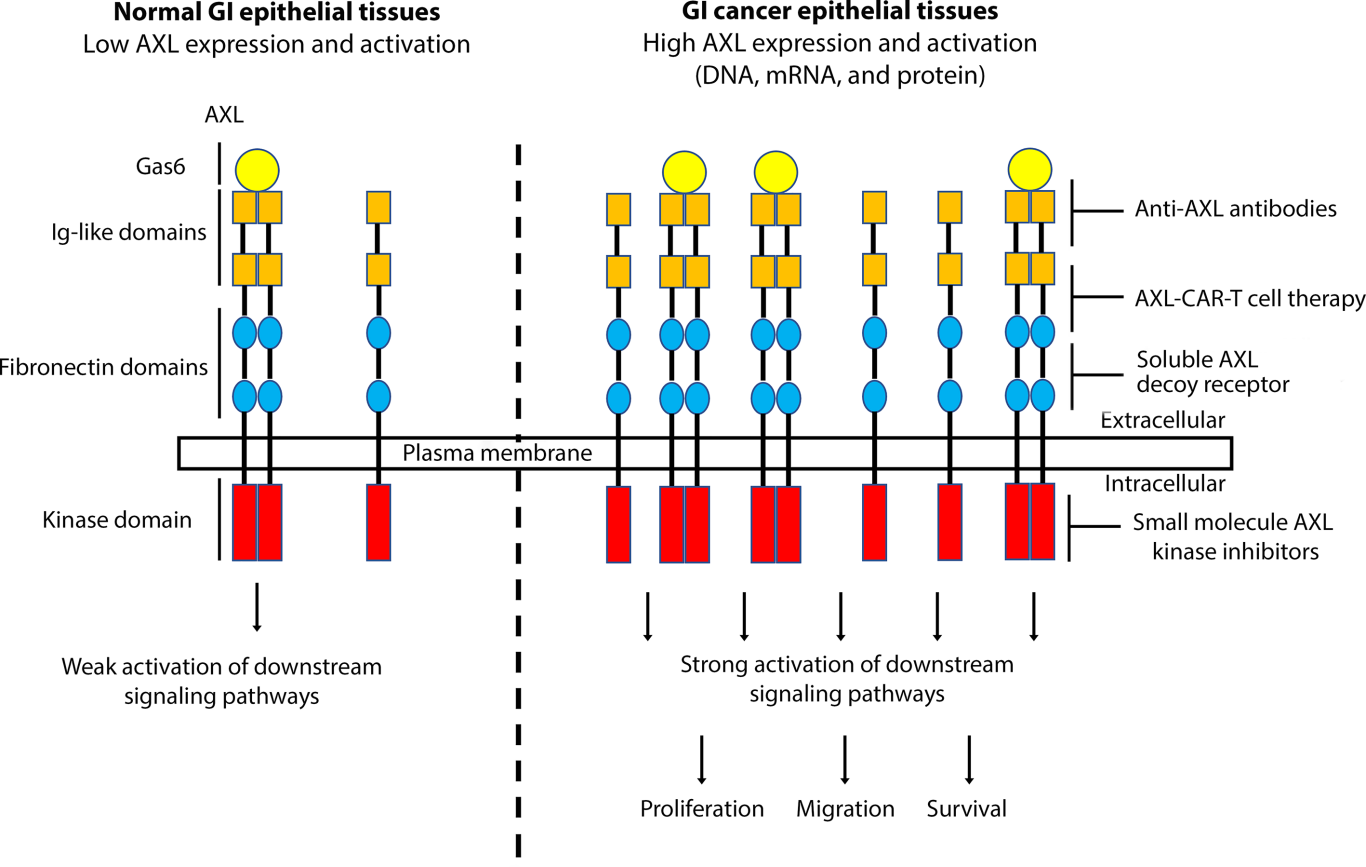
A schematic representation depicting the role of AXL overexpression and activation in GI cancers. Overexpression of AXL, induced by DNA amplification or high mRNA and protein levels, in GI epithelial tissues leads to strong activation of downstream signaling pathways, promoting cell proliferation, migration, and survival, hallmarks of GI carcinogenesis. Targeting AXL with specific monoclonal antibodies, small molecule kinase inhibitors, soluble AXL decoy receptor, or CAR-T cell therapy could be effective as a targeted therapeutic approach in GI cancers.

Collectively and based on the current literature, AXL has been associated with GI cancer development and progression and its inhibition provides a novel therapeutic approach in the fight against GI cancers (**Figure 1**). Targeting AXL alone or with other TAM receptor tyrosine kinases could stimulate antitumor immunity, reduce cancer cell survival, enhance chemosensitivity and markedly attenuate metastatic tumor burden (129, 130). AXL might potentially become a valuable therapeutic target in GI cancers, and targeted anti-AXL therapies could further improve the standard first line of therapies with the objective to improve the prognosis and clinical outcome in patients with GI cancers or other AXL-expressing tumors.

## Author contributions

NP, writing and conception of the manuscript. AB, editing the manuscript and securing the funding. All authors contributed to the article and approved the submitted version.
